# Phase stability and thermoelectric properties of semiconductor-like tetragonal FeAl_2_

**DOI:** 10.1080/14686996.2019.1662272

**Published:** 2019-09-02

**Authors:** Kazuki Tobita, Koichi Kitahara, Yukari Katsura, Naoki Sato, Daisuke Nishio-Hamane, Hirotada Gotou, Kaoru Kimura

**Affiliations:** a Department of Advanced Materials Science, The University of Tokyo, Chiba, Japan; b OPERANDO–OIL, National Institute of Advanced Industrial Science and Technology (AIST), Chiba, Japan; c International Center for Materials Nanoarchitectonics (MANA), National Institute for Materials Science (NIMS), Tsukuba, Ibaraki, Japan; d The Institute for Solid State Physics, The University of Tokyo, Chiba, Japan

**Keywords:** word, intermetallic compounds, thermoelectric materials, *in situ* X-ray diffraction, high-pressure synthesis, density functional theory, 210, 401

## Abstract

Tetragonal FeAl_2_ is a high-pressure phase and is predicted to exhibit semiconductor-like behavior. We investigated the pressure and temperature synthesizing conditions of tetragonal FeAl_2_, supported by *in situ* X-ray diffractions, using synchrotron radiation during heating the sample under a pressure of 20 GPa. Based on the determined optimal conditions, we synthesized the bulk polycrystalline samples of tetragonal FeAl_2_ at 7.5 GPa and 873 K, using a multi-anvil press and measured its thermoelectric properties. The Seebeck coefficient of tetragonal FeAl_2_ showed a large negative value of – 105 μV/K at 155 K and rapidly changed to a positive value of 75 μV/K at 400 K. Although these values are the largest among those of Fe–Al alloys, the maximum power factor remained at 0.41 mW/mK^2^ because the carrier concentration was not tuned. A comparison of the Gibbs free energy of tetragonal FeAl_2_, triclinic FeAl_2_ and FeAl+Fe_2_Al_5_ revealed that tetragonal FeAl_2_ became unstable as the temperature increased, because of its smaller contribution of vibrational entropy.

## GRAPHICAL ABSTRACT

**Figure:**
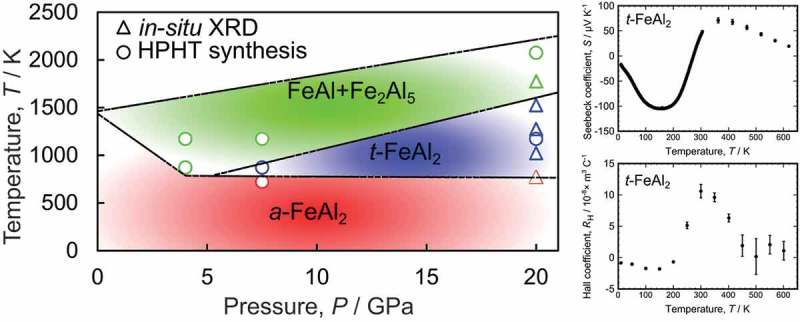

## 1. Introduction

Thermoelectric materials are functional materials that can directly convert heat into electricity. In recent years, thermoelectric materials have received a great amount of attention because they can contribute to the global energy demand by waste-heat energy harvesting. The conversion efficiency of thermoelectric materials is characterized by the dimensionless figure of merit *ZT* = *S*^2^*σT/*(*κ*_el_*+κ*_ph_), where *S, σ, κ*_el_, *κ*_ph_ and *T* are the Seebeck coefficient, electrical conductivity, electron thermal conductivity, phonon thermal conductivity and temperature, respectively. To date, most of the state-of-the-art materials like Bi_2_Te_3_ [1] and PbTe [2], which exhibit a high *ZT* of more than unity, contain toxic and/or expensive elements. For large-scale applications of thermoelectric materials, low-cost nontoxic materials with high performance are desirable. Iron aluminides are thus favorable as thermoelectric materials because of their abundance and environmentally benign constituent elements. We previously investigated the thermoelectric properties of triclinic FeAl_2_ (*a*-FeAl_2_, space group *P*−1), Fe_2_Al_5_ and Fe_4_Al_13_ in the Al-rich part of Fe–Al binary systems, and found that these materials have a low *κ*_ph_ of 0.82 W/mK (for Fe_4_Al_13_) because of their complex and disordered crystal structures [3]. However, the maximum *ZT* remained at 0.03 (for Fe_4_Al_13_) because of its low *S* of ~30 µV/K. To improve the thermoelectric properties in this binary system, semiconductors with a large magnitude of *S* and a metallic *σ* are required. However, none of the experimentally observed Fe–Al intermetallic compounds behave as semiconductors.

We previously reported that novel MoSi_2_-type tetragonal FeAl_2_ (*t*-FeAl_2_, space group *I*4/*mmm*) can be synthesized through the laser-heated diamond-anvil cell (LHDAC) technique at 10 GPa and 1873 K [4]. This *t*-FeAl_2_ was theoretically predicted to be stable (or at least metastable) [5,6], but could not be synthesized at ambient pressure [7]. Theoretical studies also predicted that *t*-FeAl_2_ exhibits a band-gap near the Fermi level, which was explained by the 14 electrons rule or 18 − *n* (*n* = 4) electrons rule, where *n* is the average number of Fe–Fe bonds [5,6,8–12]. Indeed, analog compounds RuAl_2_ and RuGa_2_ have been reported as narrow bandgap semiconductors with a high power factor, *S*^2^*σ* [7,13–16]. From these theoretical and experimental works, it is expected that *t*-FeAl_2_ also behaves like a semiconductor. However, the sample made by LHDAC was so small that the thermoelectric properties were not able to be measured. Moreover, the synthesizing temperature in the LHDAC technique has large uncertainties, because the sample volume and the infrared laser spot size are too small to accurately measure the temperature [17,18]. Therefore, the synthesizing conditions of *t*-FeAl_2_ are unclear.

In this study, to obtain the optimal conditions for synthesizing high-quality samples of *t*-FeAl_2_ under high pressure, we investigated the pressure and temperature (*P-T*) synthesizing conditions of *t*-FeAl_2_ using the multi-anvil type apparatus and *in situ* X-ray diffraction (XRD) experiments supported by synchrotron radiation. Based on the determined optimal conditions, we synthesized single-phase samples of *t*-FeAl_2_ through the high-pressure and high-temperature (HPHT) process and characterized its thermoelectric properties. Furthermore, we present a study of the stability of *t*-FeAl_2_ and neighboring phases using first-principles total energy calculations supplemented by a phonon-based calculation of vibrational entropy, which yields the Gibbs free energy under a quasi-harmonic approximation.

## 2. Experimental details

### 2.1 *In situ* x-ray diffraction experiments

The starting material powdered *a*-FeAl_2_ was prepared by arc-melting and annealing, as has been described previously [4,19]. High-pressure and high-temperature *in situ* XRD experiments were performed using multi-anvil type apparatus MAX-III installed at the AR-NE7A beamline of Photon Factory (a Synchrotron radiation facility) in the High Energy Accelerator Research Organization (KEK). The MAX-III was used as 6–8 Kawai-type multi-anvil, consisting of first-stage cubic-anvil apparatus and eight second-stage tungsten carbide cubes truncated at one corner, to accommodate an octahedral cell containing the sample and heater. The sample container and heater were made of MgO and a thin rhenium film with a thickness of 0.025 mm, respectively. Detailed cell assembly was like that described elsewhere [20]. The energy-dispersive X-ray diffraction method was adopted with a fixed Bragg angle of 2*θ =*6° in the horizontal direction. Pressure and temperature were determined by the lattice constant of a MgO internal pressure marker (following the third-order Birch–Murnaghan equation of states [21] of MgO from Ref [22].), and thermocouples installed near the sample and heater, respectively. The *in situ* XRD patterns were recorded during heating from 300 K to 1773 K at a constant pressure of 20 GPa.

### 2.2 Synthesis and characterization

Using the same starting material *a*-FeAl_2_ as used in the *in situ* XRD experiments, bulk polycrystalline samples were synthesized with two types of multi-anvil apparatus depending on the pressure range of interest: a single-stage multi-anvil was used at 4 GPa and 7.5 GPa, and a double-stage Kawai-type multi-anvil was used at 20 GPa. The heating temperatures for synthesis at 4 GPa, 7.5 GPa and 20 GPa were 873–1173 K, 723–1173 K and 1173–2073 K, respectively. The heating time was 3 h for all experiments. Phase identification was accomplished by powder XRD with Cu Kα radiation and scanning electron microscopy (SEM; JSM-6010LA, JEOL). Lattice parameters were determined with the program package RIETAN-FP [23] using silicon (NIST 640d) as an internal standard. Refinement of the atomic positions and isotropic atomic displacements was also performed with RIETAN-FP [23]. Differential scanning calorimetry (DSC; EXSTAR DSC-7020, SII Co.) was performed to investigate the thermal stability of *t*-FeAl_2_ at ambient pressure. Thermoelectric properties were characterized in the temperature range 10–600 K: *S, σ, κ* and Hall coefficient (*R*_H_) at 10–300 K were measured with a physical properties measurement system (PPMS; Quantum design) using the thermal transport option for thermoelectric properties and the AC transport option for five-wire *R*_H_; *S* at 300–600 K was measured with a ZEM-1 (ULVAC RIKO); *σ* and *R*_H_ at 300–600 K were measured with a Resitest 8300 (Toyo Technica Co.).

### 2.3 First-principles calculations

The phonon frequencies and corresponding thermodynamic properties at 0–20 GPa and 0–2000 K were calculated under the harmonic approximation by the finite displacement method [24,25] and supercell as implemented in the PHONOPY code [26]. To calculate the total energy of the supercells including displacements, we employed the plane-wave projector-augmented wave (PAW) method [27] in the framework of DFT within the generalized gradient approximation (GGA) functional in the Perdew–Burke–Ernzerhof (PBE) parameterization [28], as implemented in the Quantum ESPRESSO code [29]. We examined the initial structure of FeAl [30], *t*-FeAl_2_ [8], *a*-FeAl_2_ [31] and Fe_2_Al_5_ [32]. Since *a*-FeAl_2_ [31] and Fe_2_Al_5_ [33] contain mixed or partially occupied sites in their unit cells, we examined the following unit cells: for the two equivalent mixing sites in *a*-FeAl_2_, we examined ‘Al and Fe’ and ‘Fe and Fe’ arrangements labeled as *a*-FeAl_2_(AF) and *a*-FeAl_2_(FF), respectively (for more details, see elsewhere [4,6]); for the chains of partially occupied aluminum sites along the c-axis in Fe_2_Al_5_, we examined the experimental low-temperature tripled superlattice structure Fe_3_Al_8_-*mC*44 reported by Okamoto et al. [32], and theoretical tripled superlattice structure Fe_3_Al_8_-*oP*44 reported by Mihalkovic and Widom [6]. A 0.13 eV smearing width of the Methfessel–Paxton scheme [34] was used for all crystal structures except for *t*-FeAl_2_. Force constants were obtained from the cells whose sizes corresponded to the 3 × 3 × 3, 2 × 1 × 1, 3 × 3 × 2, 2 × 1 × 1 and 1 × 2 × 1 supercells of the primitive FeAl, *a*-FeAl_2_, *t*-FeAl_2_, Fe_3_Al_8_-*mC*44 and Fe_3_Al_8_-*oP*44 unit cells, respectively. An atomic displacement of 0.01 Å was used for all supercells. The phonon band paths were determined by using SeeK-path [35]. We used the quasi-harmonic approximation that transforms thermodynamic parameters from the function of volume (*V*) to the function of pressure (*P*) [36]. The Gibbs free energy (*G*) at the finite *P* and *T* was obtained as *G*_(*P, T*)_ = min*_V_*[*U*_el_ +*U*_ph_ + *PV* − *TS*_vib_], where *U*_el_, *U*_ph_ and *S*_vib_ are the electronic internal energy at 0 K, the phonon energy, and vibrational entropy, respectively. To compare the stabilities of *a*-FeAl_2_, *t*-FeAl_2_ and FeAl + Fe_2_Al_5_ at the stoichiometric composition of *t*-FeAl_2_, we used 1/3 × *G*(*a*-FeAl_2_(FF)) + 2/3 × *G*(*a*-FeAl_2_(AF)) and 4/15 × *G*(FeAl) + 11/15 × *G*(Fe_3_Al_8_) as *G*(*a*-FeAl_2_) and *G*(FeAl + Fe_2_Al_5_), respectively, for nomalization. This normalization of composition is graphically explained in Fig. S1.

## 3. Results and discussion

### 3.1 *Sample preparation and* P-T *synthesizing conditions of feal_2_*

**Figure 1.**
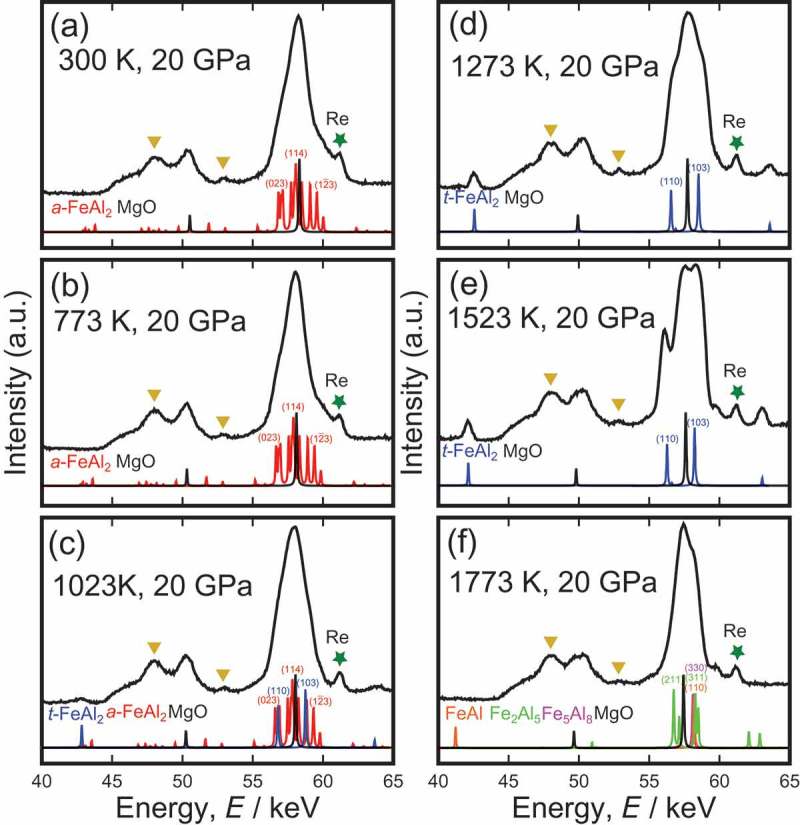
*In situ* X-ray diffraction patterns of *a*-FeAl_2_ at 20 GPa and (a) 300 K, (b) 773 K, (c) 1023 K, (d) 1273 K, (e) 1523 K and (f) 1773 K. Solid stars and triangles indicate the characteristic X-ray for rhenium and unidentified peaks derived from something outside the sample container, respectively. *t*-FeAl_2_ was observed between 1073 K and 1523 K, but disappeared at 1773 K. The intensity is plotted on a log scale.

Figure 1 shows *in situ* XRD patterns (log scale) of FeAl_2_ at 20 GPa and 300–1773 K. The Bragg peaks of MgO (sample container) and characteristic X-ray peaks of rhenium (sample heater) were observed at all temperatures. The peaks represented by triangle symbols did not shift even when temperature increased, suggesting these peaks were derived from something outside sample container. Although the weak and broad peaks of the starting material *a*-FeAl_2_ were observed at 300–1023 K, we observed the peaks of *t*-FeAl_2_, where the intensity increased, and the peak sharpened as the temperature increased at 1023 K – 1523 K. However, the peaks of *t*-FeAl_2_ disappeared at 1773 K, which indicated *t*-FeAl_2_ was unstable. Instead of the peaks of *t*-FeAl_2_, weak peaks similar to those of the neighboring phases FeAl, Fe_5_Al_8_ (high-temperature phase) and Fe_2_Al_5_ wereobserved. Unfortunately, the strong peaks of FeAl, Fe_5_Al_8_ and Fe_2_Al_5_ were so close to those of MgO that it was difficult to determine which phases appeared at 1773 K.

**Figure 2.**
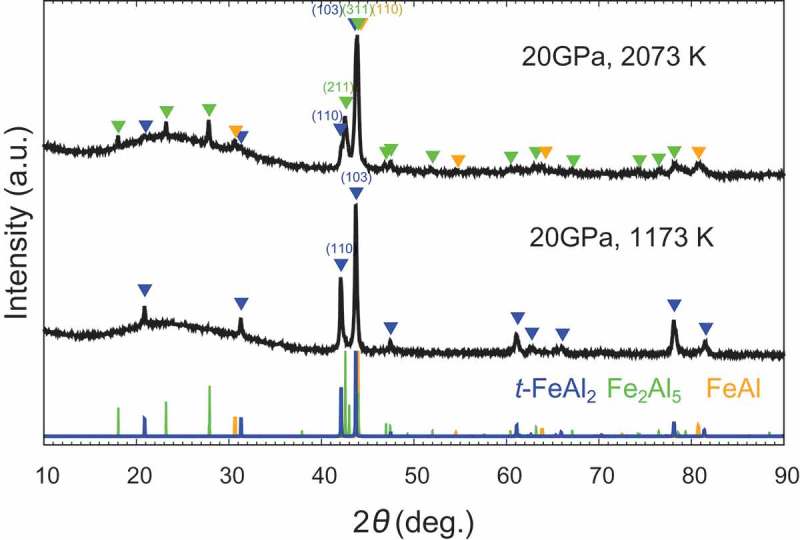
X-ray diffraction patterns with Cu Kα of the sample synthesized at (top) 20 GPa and 2073 K and (bottom) 20 GPa and 1173 K. We succeeded in synthesizing a single-phase *t*-FeAl_2_ sample at 20 GPa and 1173 K, whereas most of the *t*-FeAl_2_ decomposed into FeAl and Fe_2_Al_5_ at 20 GPa and 2073 K.

**Figure 3.**
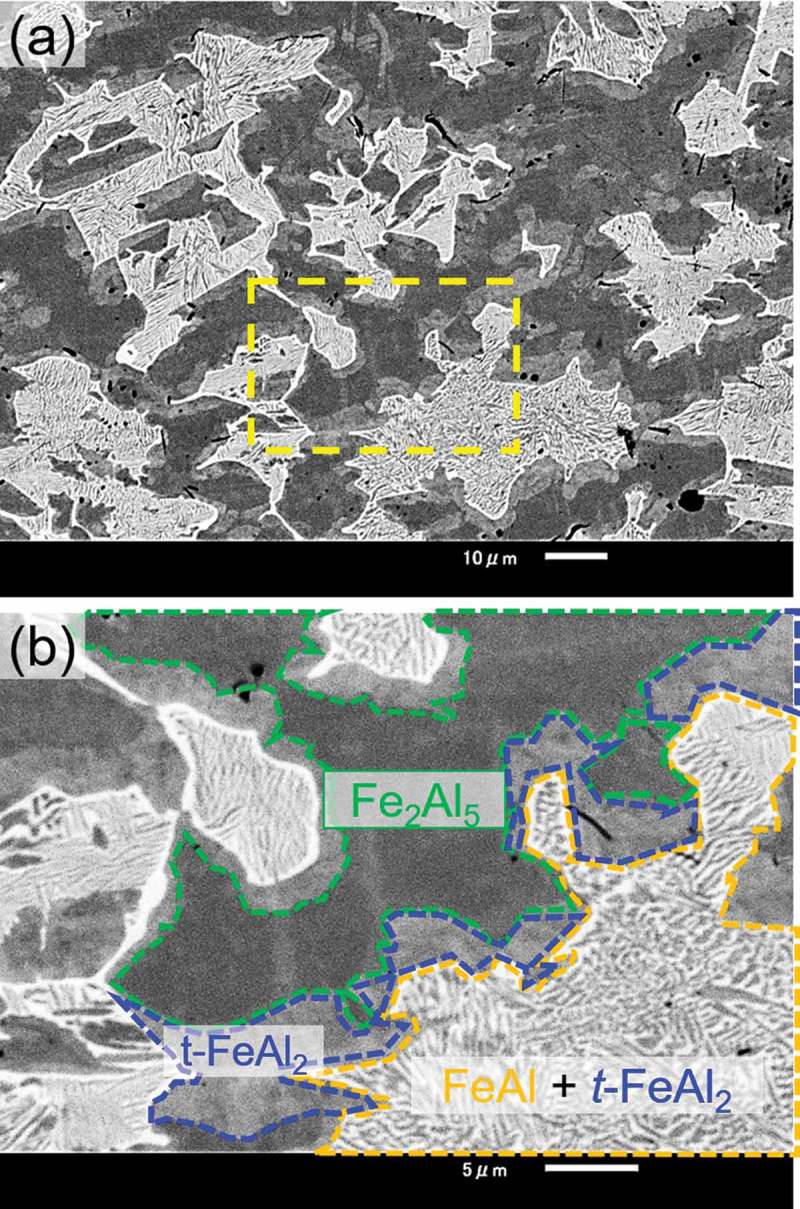
(a) SEM-BECI image of the sample synthesized at 20 GPa and 2073 K. (b) Magnified image shows the growth of *t*-FeAl_2_ at phase boundaries between a lamellar matrix of FeAl + *t*-FeAl_2_ and Fe_2_Al_5._

**Figure 4.**
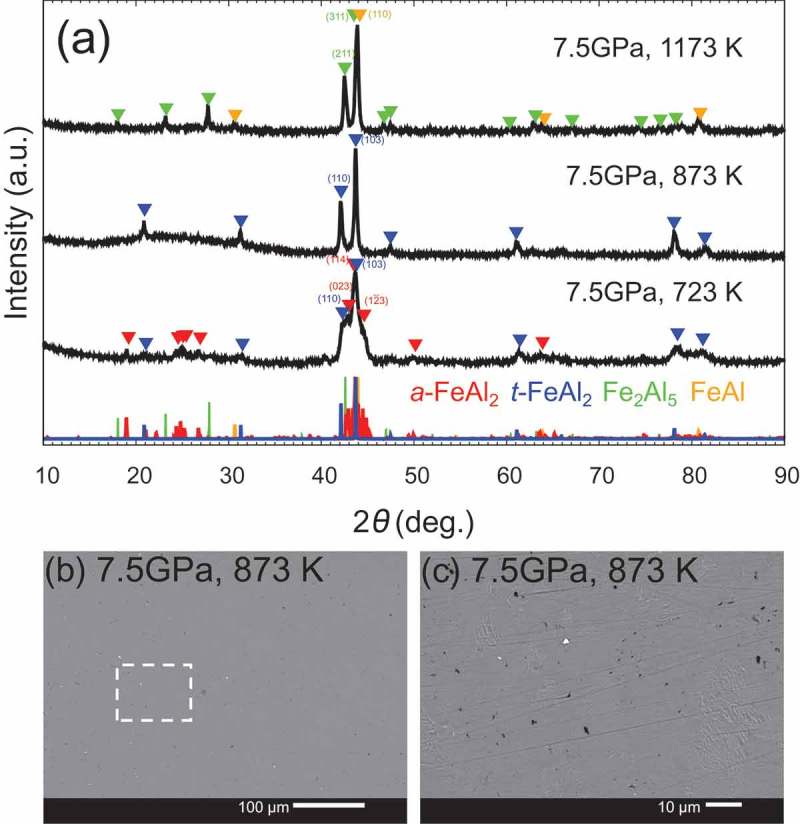
(a) X-ray diffraction patterns with Cu Kα of the sample synthesized at (top) 7.5 GPa and 1173 K (middle) 7.5 GPa and 873 K, and (bottom) 7.5 GPa and 723 K. *t*-FeAl_2_ was successfully synthesized at 7.5 GPa and 873 K, but completely decomposed at 1173 K. The *a*-FeAl_2_ was retained in the sample synthesized at 7.5 GPa and 723 K because the heating time for the HPHT process was limited to 3 h. (b) and (c) SEM-BECI image of the sample synthesized at 7.5 GPa and 873 K, which shows small impurities in the sample.

**Figure 5.**
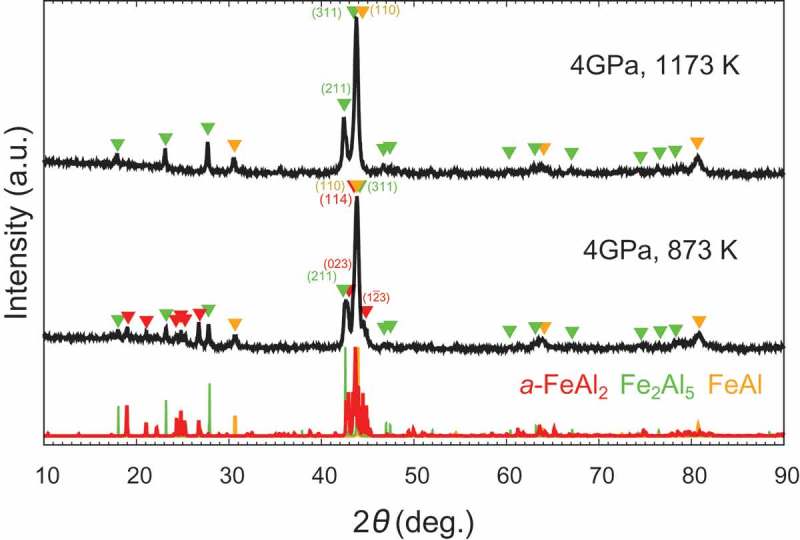
X-ray diffraction patterns with Cu Kα of the sample synthesized at (top) 4 GPa and 1173 K, and (bottom) 4 GPa and 873 K. The direct decomposition from *a*-FeAl_2_ to FeAl and Fe_2_Al_5_ was observed at 4 GPa and 873 K. *t*-FeAl_2_ was not observed over the entire temperature range.

Based on the results of the *in situ* observation, HPHT synthesis was performed at 20 GPa. Figure 2 shows the XRD patterns with Cu Kα of the samples synthesized at 20 GPa. We successfully synthesized *t*-FeAl_2_ at 1173 K, whereas the sample recovered from the run at 2073 K mainly decomposed to FeAl and Fe_2_Al_5_. The presence of *t*-FeAl_2_ at 1173 K and mostly its absence at 2073 K were in good agreement with the *in situ* XRD observation. The backscattered electron composition image (BECI) of the sample synthesized at 20 GPa and 2073 K showed three kinds of regions: FeAl (light contrast), *t*-FeAl_2_ (gray) and Fe_2_Al_5_ (dark) as shown in Figure 3. We observed a lamellar matrix of FeAl and *t*-FeAl_2_, and *t*-FeAl_2_ at the phase boundaries between the lamellar matrix and Fe_2_Al_5_. X. Li et al. observed the similar microstructure in a levitation melted Fe_33.2_Al_66.8_ at ambient pressure, and explained the microstructure by the eutectic reaction of the high-temperature phase Fe_5_Al_8_ into FeAl and *a*-FeAl_2_ [19]. Assuming the same situation at high pressure except for replacing *a*-FeAl_2_ with *t*-FeAl_2_, the microstructure in Figure 3 is well explained as follows: the sample formed Fe_5_Al_8_ and Fe_2_Al_5_ at 2073 K, and then *t*-FeAl_2_ grew peritectoidally between Fe_5_Al_8_ and Fe_2_Al_5_ during cooling process; in a later stage of cooling, Fe_5_Al_8_ decomposed into FeAl and *t*-FeAl_2_ by eutectoid reaction. Therefore, *t*-FeAl_2_ was suggested to be unstable and decomposed at 2073 K. To obtain the large bulk samples, we attempted to synthesize the samples at lower pressure. Figures 4(a) and 5 show XRD patterns of the sample synthesized at 4 GPa and 7.5 GPa, respectively. We successfully synthesized single-phase *t*-FeAl_2_ at 7.5 GPa and 873 K, but we observed small impurities in the BECI shown in Figure 4(b)–(c). The composition of these small impurities, Fe_36_Al_64_ (light contrast) and Fe_32_Al_68_ (dark), was slightly different from FeAl_2_. Since we did not observe any peaks derived from these impurities, the amount of the impurities should be low. With the increasing temperature at 7.5 GPa, the peak of *t*-FeAl_2_ faded, and those of FeAl and Fe_2_Al_5_ were observed. When we synthesized the samples at 4 GPa, *t*-FeAl_2_ was not observed at all in the temperature range and direct decomposition from *a*-FeAl_2_ to FeAl and Fe_2_Al_5_ was observed at 873 K.

**Figure 6.**
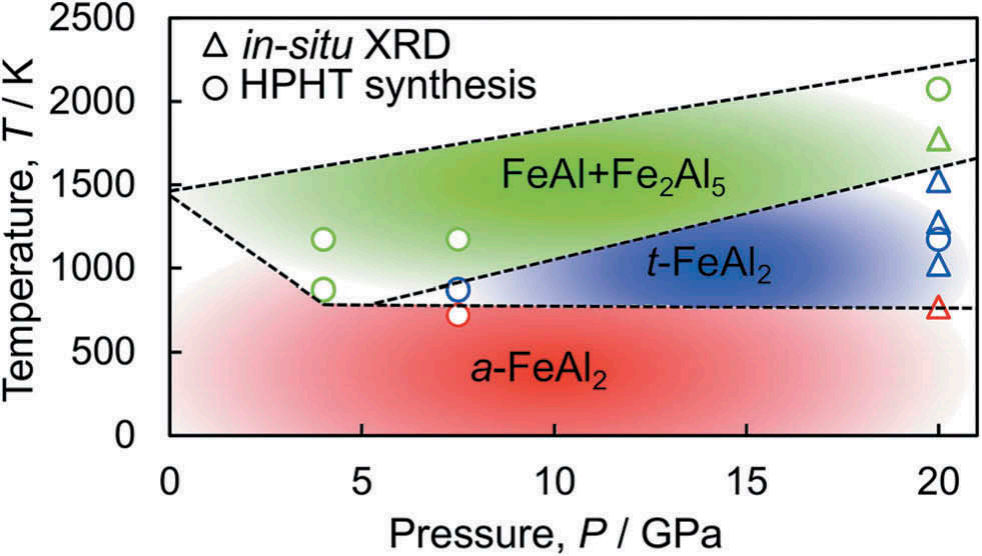
Pressure and temperature synthesizing conditions of FeAl_2_ determined by the *in situ* XRD and quenching experiments using *a*-FeAl_2_ as a starting material. As the pressure increases, the temperature range at which *t*-FeAl_2_ is stable expands monotonically. However, further increase of temperature results in the decomposition to FeAl and Fe_2_Al_5_. Note that the results at low temperature may change if heating time is extended because heating time was as short as 3 h.

We summarized the series of experiments as a *P-T* synthesizing conditions (not an equilibrium phase diagram, but merely shows the phases observed after HPHT synthesis) of FeAl_2_ using *a*-FeAl_2_ as a starting material, as is shown in Figure 6. We have to note that these results may change with starting materials and heating time. According to the phase diagram at ambient pressure, *a*-FeAl_2_ was stable until the temperature reached 1418 K [19]. However, the decomposition or phase transition of *a*-FeAl_2_ was observed at a lower temperature when subjected to a higher pressure. This trend was in good agreement with the enthalpy calculation where *a*-FeAl_2_ became less stable than *t*-FeAl_2_ and FeAl + Fe_2_Al_5_ at high pressure [4]. One can see that as the pressure increased from 7.5 GPa to 20 GPa, the temperature range where *t*-FeAl_2_ was stable expanded monotonically. However, with a further increase in temperature, *t*-FeAl_2_ decomposed into FeAl and Fe_2_Al_5_ over the entire pressure range. When we synthesized *t*-FeAl_2_ by LHDAC technique, the temperatures (1873 K at 10 GPa and 2123 K at 20 GPa) were higher than our present study [4]. One possible explanation for this discrepancy may be the inhomogeneity during the heating process in our previous LHDAC samples. Since the diameter of heating laser (10 μm) was much smaller than sample diameter (100 μm), the temperature outside the laser lower may be lower than the measured temperature, resulting in we observed *t*-FeAl_2_ at such high temperatures. Therefore, we did not include the results of LHDAC samples in Figure 6.

Table 1 shows the lattice parameters obtained with an internal silicon standard, and the atomic positions and isotropic atomic displacements of *t*-FeAl_2_ synthesized at 7.5 GPa and 873 K. The lattice parameters (*a* = 3.0307(1) Å^3^ and *c* = 8.4959(3) Å^3^) were in good agreement with those calculated in previous reports within 1.5% [4,8]. The corresponding atomic volume of 13.0 Å^3^/atom was denser than the 13.8 Å^3^/atom of *a*-FeAl_2_ [31], which indicated that *t*-FeAl_2_ became more stable than *a*-FeAl_2_ as the pressure increased. In the assigned space group *I*4/*mmm*, only the *z* position of Al atoms was refined to be *z* = 0.3429(3).

**Table 1.** Results of structure refinement for *t*-FeAl_2._

Crystal data		
Crystal system	Tetragonal	
Space group, Pearson symbol	*I*4/*mmm*, tI6	
*a*(Å)	3.0307(1)	
*c*(Å)	8.4959(3)	
Atomic parameters		
Atom	Fe	Al
Wyckoff notation	2a	4e
Symmetry	4/*mmm*	4*mm*
*x*	0	0
*y*	0	0
*z*	0	0.3429(3)
*U*_iso_ (Å^2^)	0.0071(8)	0.0043(8)
Reliability factors		
*R*_wp_	4.63%	
*R*_p_	3.59%	
*R*_B_	6.77%	
*R*_F_	6.15%	
*S*	1.79	

**Figure 7.**
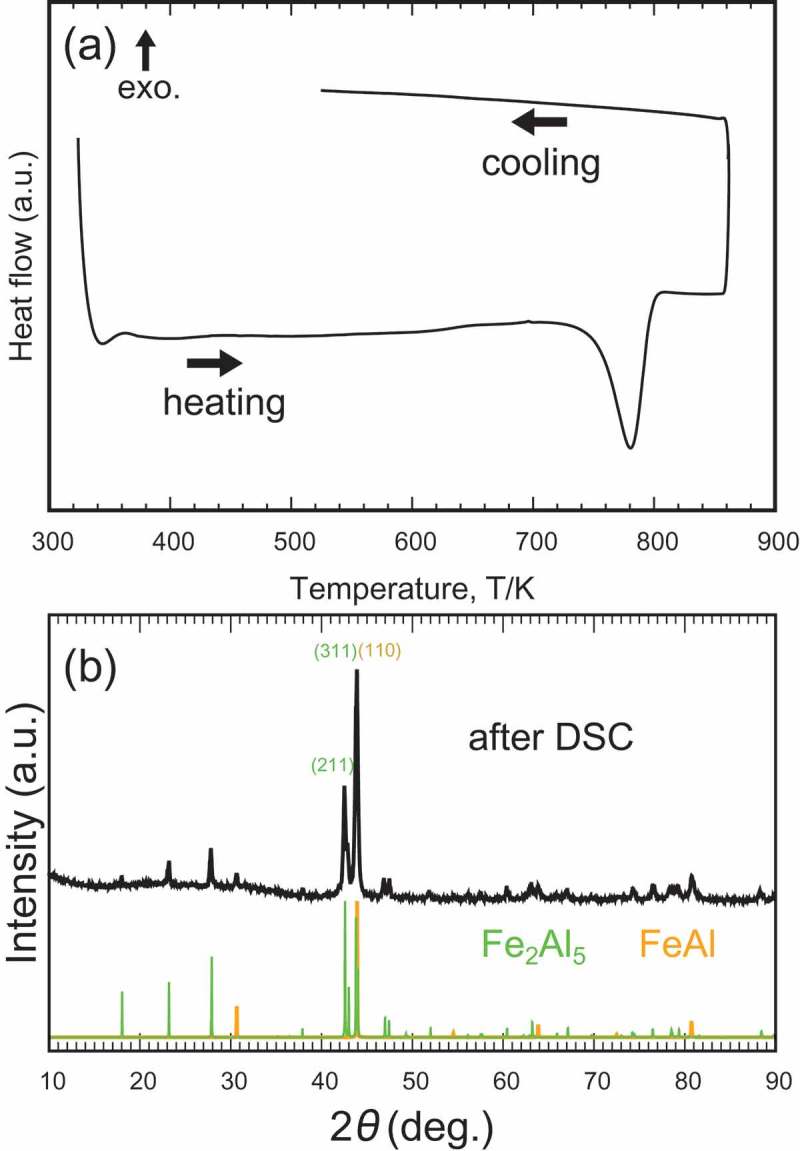
(a) DSC measurement with the endothermic signal at 787 K, which indicates decomposition of *t*-FeAl_2_. (b) X-ray diffraction patterns of *t*-FeAl_2_ after DSC measurement, which results in the complete decomposition into FeAl and Fe_2_Al_5._

When *t*-FeAl_2_ was heated at ambient pressure, *t*-FeAl_2_ decomposed into FeAl and Fe_2_Al_5_, as observed from the strong endothermic signal in the DSC measurement at around 787 K during heating (see Figure 7(a)). Integration of the endothermic peak shows that *t*-FeAl_2_ is more stable than FeAl + Fe_2_Al_5_ by 11 meV/atom in terms of the formation enthalpy. However, in this study, we did not observe the phase transition during cooling process, suggesting the transition temperature during cooling process may be lower than the temperature during heating process, and/or atomic diffusion may be so slow that we cannot observe it. The powdered XRD patterns showed the complete absence of *t*-FeAl_2_ after DSC measurements (see Figure 7(b)). This decomposition seemed to be strange since *a*-FeAl_2_ is stable below 1418 K from the phase diagram [19], but it could be explained by the fact that the nucleation and/or growth of *a*-FeAl_2_ was much slower than that of Fe_2_Al_5_ at 823–913 K [37].

### 3.2 Thermoelectric properties of feal_2_

**Figure 8.**
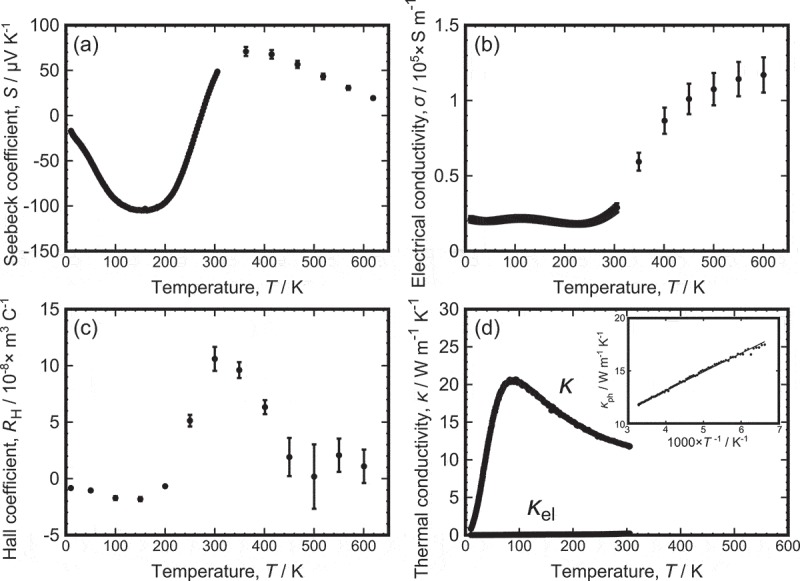
Thermoelectric properties of pristine *t*-FeAl_2_: (a) Seebeck coefficient, (b) electrical conductivity, (c) Hall coefficient and (d) thermal conductivity. The inset of (d) shows phonon thermal conductivity versus 1000/*T* plot.

Figure 8(a)–(d) shows the temperature dependence of *S, σ, R*_H_ and total thermal conductivity (*κ*), respectively. The standard uncertainty was calculated by considering the uncertainty in the sample dimensions and instruments. We observed a semiconductor-like large *S*, which rapidly changed from a negative value of −105 μV/K (at 155 K) to a positive value of 75 μV/K (at 400 K). Our previous calculation [4] showed that the density of states (DOS) at the conduction band minimum was higher than that at the valence band maximum, which was in good agreement with the larger *S* observed in the n-type. The *S* decreased above 400 K since the estimated bandgap of *t*-FeAl_2_ was as narrow as 0.035 eV [4]. The major carrier type change from negative to positive was well reproduced in the *R*_H_. The *σ* was kept almost constant up to 250 K, which was approximately the sign change temperature of *R*_H_ and *S*, and began to increase to ~1.3 × 10^5^ S/m. The *κ*_el_ was estimated using the measured *σ* in conjunction with the Wiedemann–Franz law at 10–300 K, which showed that *κ*_el_ was less than 0.2 W/mK over the entire temperature range (See Figure 8(d)). The *κ*_ph_ is written as follows by applying the phonon gas theory:

(1) $$\kappa_{ph}=\frac{1}{3}C_{v}\tau^{2}v$$

where *C*_v_, *τ* and *v* are the heat capacity, phonon relaxation time and average phonon group velocity, respectively. The increase in *κ*_ph_ with increasing *T* below 80 K mainly arose from the increase of *C*_v_, whereas the reduction in *κ*_ph_ above 80 K resulted from a decrease in *τ*. As shown in the inset of Figure 8(d), the temperature dependence of *κ*_ph_ from 150 K to 300 K was proportional to 1/*T*, which suggested that Umklapp scattering played the main role in the decrease of *κ*_ph_. One can compare the thermoelectric properties of *t*-FeAl_2_ with those of other intermetallic thermoelectrics, Fe_2_VAl [38–42] and Al_2_Fe_3_Si_3_ [43–46]. The *κ*_ph_ of *t*-FeAl_2_ was several times larger than that of Al_2_Fe_3_Si_3_. The maximum *S* of *t*-FeAl_2_ was comparable to those of Fe_2_VAl and Al_2_Fe_3_Si_3_, but *σ* of *t*-FeAl_2_ was approximately one order of magnitude smaller than Fe_2_VAl. Therefore, the maximum power factor *S*^2^*σ* remained as low as ~0.41 mW/mK^2^ (at 400 K). The poor *S*^2^*σ* and large *κ* led to a low maximum ZT of 2.3 × 10^−3^ (at 180 K). However, we previously predicted that *t*-FeAl_2_ will have a larger power factor if n-type doping is possible [4]. Thus, further research concerning carrier tuning will enhance the thermoelectric properties of *t*-FeAl_2_.

### 3.3 Phonon calculations

**Figure 9.**
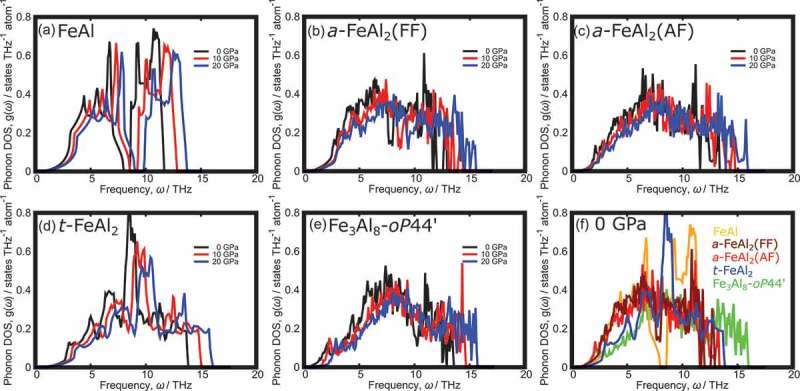
Phonon density of states (DOS) of (a) FeAl, (b) *a*-FeAl_2_(FF), (c) *a*-FeAl_2_(AF), (d) *t*-FeAl_2_, (e) Fe_3_Al_8_-*oP*44ʹ and (f) all structures at 0 GPa. The phonon DOS of a-FeAl_2_ and Fe_2_Al_5_ show enhanced low-frequency phonons, which can contribute to vibrational entropy.

**Figure 10.**
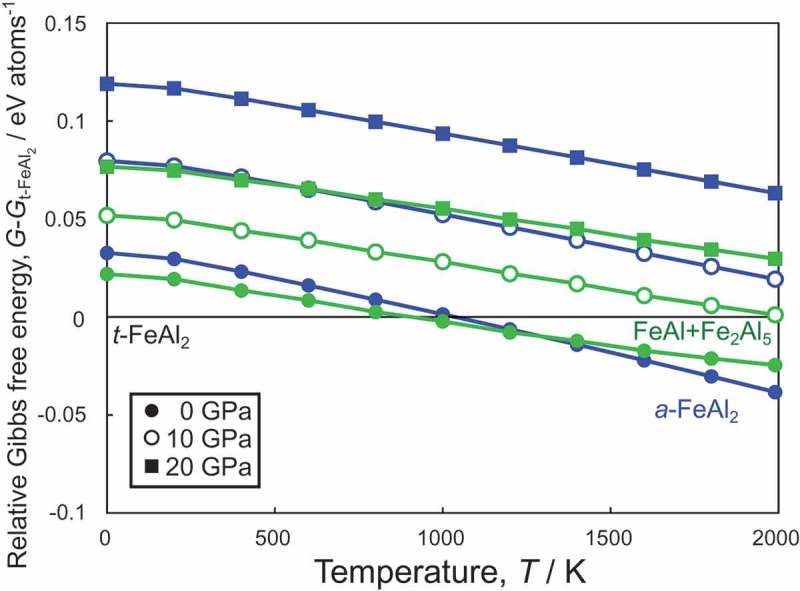
Relative Gibbs free energies (with respect to *t*-FeAl_2_) of (blue line) *a*-FeAl_2_, and (green line) FeAl + Fe_2_Al_5_ at (solid circle) 0 GPa (open circle) 10 GPa, and (solid square) 20 GPa as a function of temperature. As temperature increases, *a*-FeAl_2_ and FeAl + Fe_2_Al_5_ tune to be more stable than *t*-FeAl_2_. At high pressures and high temperatures, FeAl + Fe_2_Al_5_ seems to be the most stable since the contribution of *TS*_vib_ in FeAl + Fe_2_Al_5_ is slightly inferior to *a*-FeAl_2_, but FeAl + Fe_2_Al_5_ is more stabilized than *a*-FeAl_2_ in terms of *PV.*

We found the phonon band structure of Fe_3_Al_8_-*oP*44 shows imaginary phonon modes at gamma points (see Fig. S2(a)). However, the imaginary phonon modes were disappeared by shifting the atomic coordinates of Fe_3_Al_8_-*oP*44 in the direction of the eigenvector where the imaginary phonon modes appeared (see Fig. S2). Tables S1–S6 show the structural parameters of all structures obtained by the structural relaxation including the shifted Fe_3_Al_8_-*oP*44 as labeled Fe_3_Al_8_-*oP*44ʹ. Since our total energy calculation revealed that the shifted structure labeled as Fe_3_Al_8_-*oP*44ʹ is slightly more stable than Fe_3_Al_8_-*mC*44 by 2.2 meV/atoms, we used Fe_3_Al_8_-*oP*44ʹ in the following Gibbs energy calculations. The phonon DOS of FeAl, *a*-FeAl_2_, *t*-FeAl_2_ and Fe_3_Al_8_-*oP*44ʹ at 0–20 GPa are shown in Figure 9, respectively. All of the structures exhibited no imaginary phonon modes, which indicated these structures were dynamically stable even at high pressure. With increasing pressure, the phonon frequencies shifted to high energy, whereas the shape of the phonon DOS was retained. The phonon contributions for Helmholtz free energy, *F*_vib_, is obtained from the following equation:

(2) $$F_{vib}=\;\frac{1}{2}\sum_{qv}\hbar w\left(qv\right)-k_{B}T\sum_{qv}ln{\left(1-exp\left(-\frac{\hbar w\left(qv\right)}{k_{B}T}\right)\right)}^{-1}$$

where *w*(*qv*) are phonon frequencies of band index *v* at each *q*-point. The lower-frequency phonons make the second term of Eq. (2) larger, contributing to the stability at high temperature. Figure 9(f) shows *a*-FeAl_2_ and Fe_3_Al_8_-*oP*44ʹ exhibit the larger density of low-frequency phonons than other structures, suggesting these two structures will be more stabilized at high temperature. Figure 10 shows the temperature dependence of the relative Gibbs free energies of *a*-FeAl_2_ and FeAl + Fe_2_Al_5_ with respect to *t*-FeAl_2_. When the pressure increased, *PV* shifted in the direction of the stabilization of *t*-FeAl_2_ due to its denser structure, whereas *TS*_vib_ was almost constant at various pressures. Owing to the small contribution of *TS*_vib_ in *t*-FeAl_2_, *t*-FeAl_2_ became unstable with respect to FeAl + Fe_2_Al_5_ and *a*-FeAl_2_ as the temperature increased. The most stable phase at 0GPa and low temperature was *t*-FeAl_2_ in our calculations, which is in good agreement with the DSC measurement. Our calculations show the formation enthalpy difference between *t*-FeAl_2_ and FeAl + Fe_2_Al_5_ is 22 meV/atoms, which is slightly larger than the DSC measurement. It was suggested that FeAl + Fe_2_Al_5_ was most stable at high temperatures and high pressures since the contribution of *TS*_vib_ in FeAl + Fe_2_Al_5_ was slightly inferior to *a*-FeAl_2_, but FeAl + Fe_2_Al_5_ was more stable than *a*-FeAl_2_ in terms of *PV*. Comparing the *P-T* synthesizing conditions obtained from the experiment with these calculations, unfortunately, the phase transition temperature was overestimated from those investigated in the experiment. The difficulty in reproducing the atomic disorders in *a*-FeAl_2_ and Fe_2_Al_5_ in the primitive cells results in our calculations underestimated the Gibbs free energies of these structures. However, the trends that (i) the stable temperature range of *t*-FeAl_2_ expanded as pressure increased, and (ii) FeAl + Fe_2_Al_5_ became more stable than *a*-FeAl_2_ and *t*-FeAl_2_ at high temperature and high pressure, were well reproduced in the calculations.

## 4. Conclusions

In this study, using *a*-FeAl_2_ as the starting material, the *P-T* synthesizing conditions of *t*-FeAl_2_ were determined for the first time. Supported by *in situ* XRD measurements, we found *t*-FeAl_2_ was synthesized at 7.5 and 20 GPa. However, by increasing the temperature to 1173 K (at 7.5 GPa) and 1773 K (at 20 GPa), *t*-FeAl_2_ decomposed into the neighboring phases FeAl and Fe_2_Al_5_. The *S* of *t*-FeAl_2_ is −105 μV/K (at 150 K), which is the largest value in a Fe–Al binary system, however, the maximum power factor is retained as 0.41 mW/mK^2^ because the carrier concentration is not tuned. The calculated phonon DOS for FeAl, *a*-FeAl_2_, *t*-FeAl_2_ and Fe_2_Al_5_ shows no imaginary frequencies over the entire pressure range, which indicates these phases are dynamically stable. A comparison of the *G* of FeAl, *a*-FeAl_2_, *t*-FeAl_2_ and Fe_2_Al_5_ reveals that (i) although *t*-FeAl_2_ is stable at high pressure owing to the *PV, t*-FeAl_2_ becomes unstable as the temperature increases, and (ii) FeAl + Fe_2_Al_5_ is most stable at high pressure and high temperature. Although the phase transition and decomposition temperature were overestimated from those obtained from experiment, these results explain the trends we observe in the experiments.
