# Supplementary material for: Phase stability and thermoelectric properties of semiconductor-like tetragonal FeAl2
Source: Sci Technol Adv Mater. 2019 Sep 2;20(1):937–48. doi: 10.1080/14686996.2019.1662272 (PMC6764376; doi:10.1080/14686996.2019.1662272)
Supplement: Supplemental Material [file TSTA_A_1662272_SM5924.docx]

SUPPLMENTAL MATERIAL FOR:

Phase stability and thermoelectric properties of semiconductor-like tetragonal FeAl_2_

Kazuki Tobita^a,^*, Koichi Kitahara^a,b^, Yukari Katsura^a^, Naoki Sato^c^, Daisuke Nishio-Hamane^d^, Hirotada Gotou^d^_,_ and Kaoru Kimura^a,b^

^a^Department of Advanced Materials Science, The University of Tokyo, 5-1-5 Kashiwanoha, Kashiwa, Chiba 277-8561, Japan

^b^OPERANDO–OIL, National Institute of Advanced Industrial Science and Technology (AIST), 148 City Block 4, Kashiwanoha Campus, 178-4 Wakashiba, Kashiwa, Chiba 277-0871, Japan

^c^International Center for Materials Nanoarchitectonics (MANA), National Institute for Materials Science (NIMS), 1-1 Namiki, Tsukuba, Ibaraki, 305-0044, Japan

^d^The Institute for Solid State Physics, The University of Tokyo, 5-1-5 Kashiwanoha, Kashiwa, Chiba 277-8581, Japan

*Corresponding Author.

E-mail: [t.kazuki52@phys.mm.t.u-tokyo.ac.jp](mailto:t.kazuki52@phys.mm.t.u-tokyo.ac.jp)

Phone/Fax: +81-4-7136-3759

Address: Kibantoh 502, 5-1-5 Kashiwanoha, Kashiwa, Chiba 277-8561, Japan


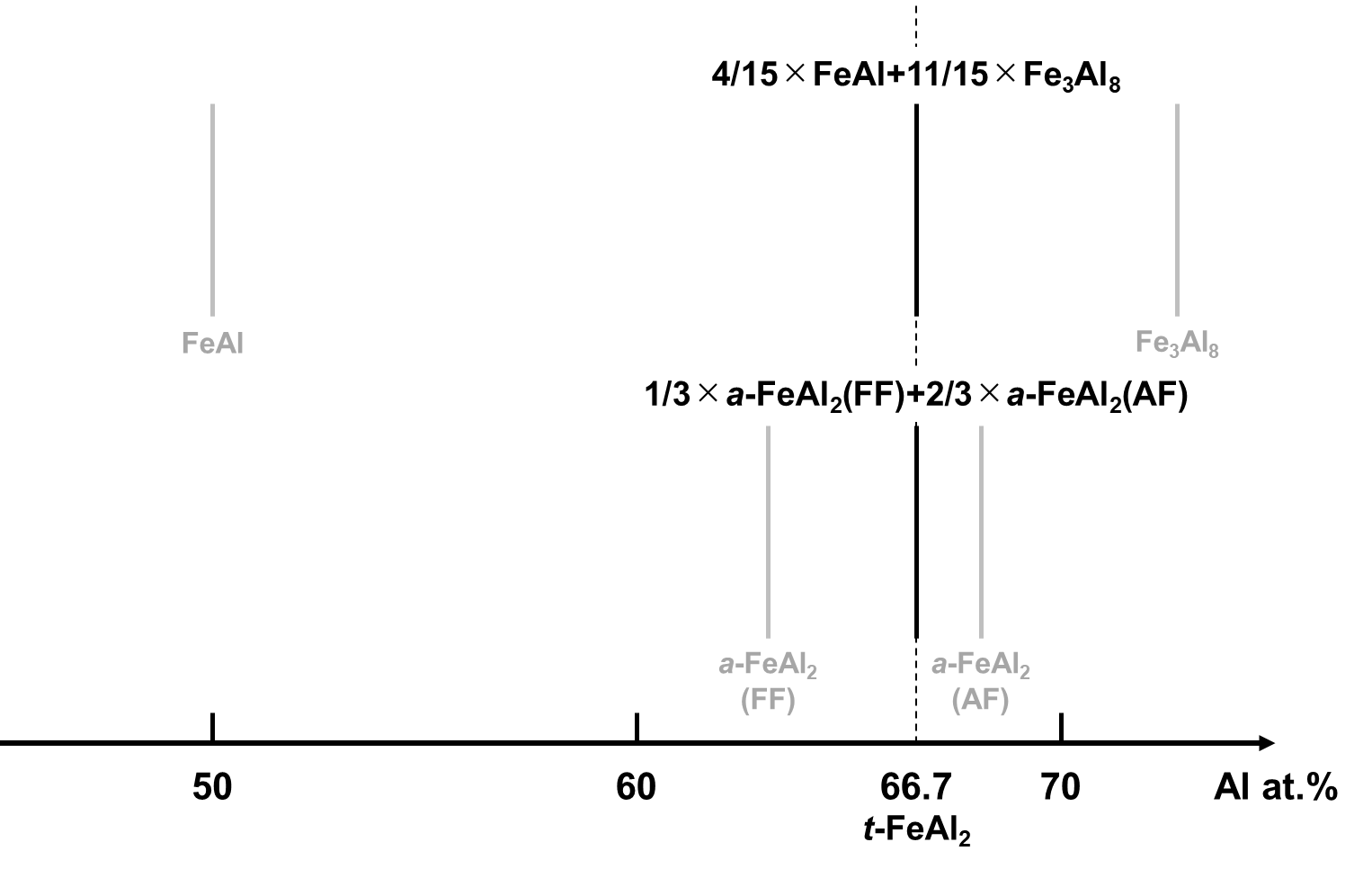


**Fig. S1.** The normalization of composition to comparing the Gibbs free energies at the same composition (Al 66.7at.%).


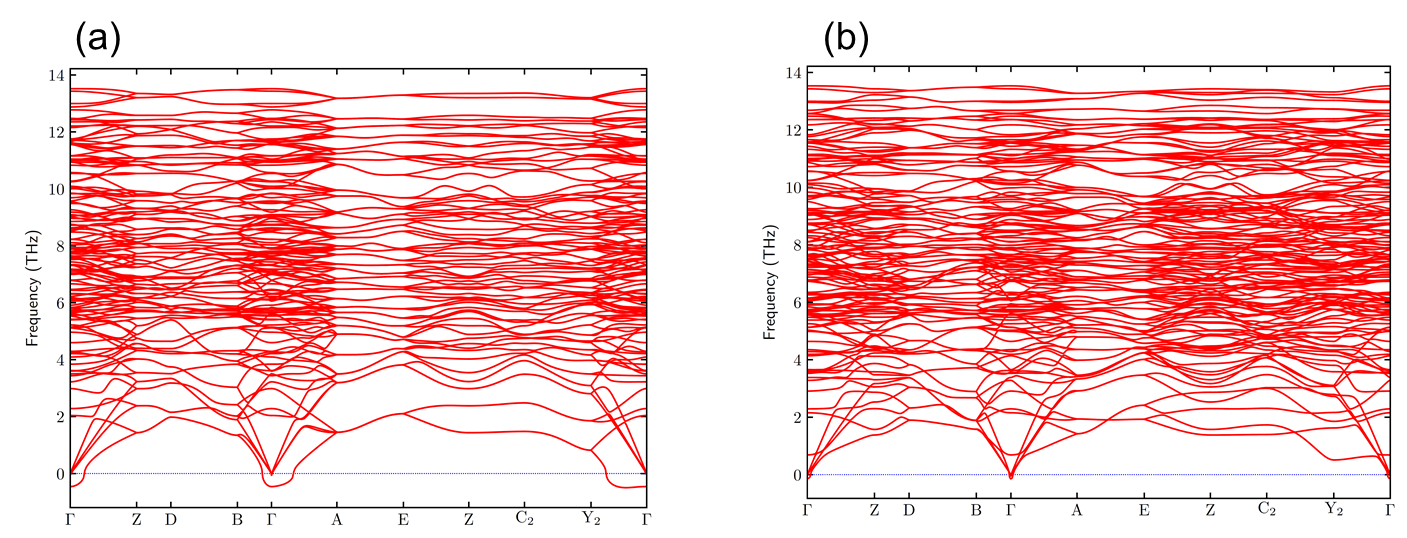


**Fig. S2.** The phonon band structures of (a) Fe_3_Al_8_-oP*44* and (b) its refined structure　Fe_3_Al_8_-*oP*44’. The imaginary phonon modes at gamma point were disappeared by shifting the atomic coordinates of Fe_3_Al_8_-*oP*44 in the direction of the eigenvector where the imaginary phonon modes appeared. The band-path was determined by using SeeK-path [1].

**Table S1**. The structural parameters of FeAl primitive cell obtained by the structural relaxation.

| Lattice parameters |  |  |  |
| --- | --- | --- | --- |
| *a*(Å) | 2.875 | | |
| *b*(Å) | 2.875 | | |
| *c*(Å) | 2.875 | | |
| *α* | 90.000 | | |
| *β* | 90.000 | | |
| *γ* | 90.000 | | |
| Atomic coodinates | *x* | *y* | *z* |
| Fe1 | 0.500 | 0.500 | 0.500 |
| Al1 | 0.000 | 0.000 | 0.000 |

**Table S2.** The structural parameters of t-FeAl_2_ primitive cell obtained by the structural relaxation.

| Lattice parameters |  |  |  |
| --- | --- | --- | --- |
| *a*(Å) | 3.003 | | |
| *b*(Å) | 3.003 | | |
| *c*(Å) | 4.750 | | |
| *α* | 71.572 | | |
| *β* | 71.572 | | |
| *γ* | 90.000 | | |
| Atomic coodinates | *x* | *y* | *z* |
| Fe1 | 0.000 | 0.000 | 0.000 |
| Al1 | 0.653 | 0.653 | 0.693 |
| Al2 | 0.347 | 0.347 | 0.307 |

**Table S3**. The structural parameters of a-FeAl_2_(FF) primitive cell obtained by the structural relaxation.

| Lattice parameters |  |  |  |
| --- | --- | --- | --- |
| *a*(Å) | 4.803 | | |
| *b*(Å) | 6.344 | | |
| *c*(Å) | 8.785 | | |
| *α* | 87.523 | | |
| *β* | 74.498 | | |
| *γ* | 83.644 | | |
| Atomic coodinates | *x* | *y* | *z* |
| Fe1 | 0.143 | 0.155 | 0.418 |
| Fe2 | 0.857 | 0.845 | 0.582 |
| Fe3 | 0.220 | 0.345 | 0.888 |
| Fe4 | 0.780 | 0.655 | 0.112 |
| Fe5 | 0.000 | 0.000 | 0.000 |
| Fe6 | 0.166 | 0.478 | 0.583 |
| Fe7 | 0.834 | 0.522 | 0.417 |
| Al1 | 0.488 | 0.013 | 0.169 |
| Al2 | 0.512 | 0.987 | 0.831 |
| Al3 | 0.039 | 0.129 | 0.708 |
| Al4 | 0.961 | 0.871 | 0.292 |
| Al5 | 0.601 | 0.186 | 0.514 |
| Al6 | 0.399 | 0.814 | 0.486 |
| Al7 | 0.004 | 0.296 | 0.178 |
| Al8 | 0.996 | 0.704 | 0.822 |
| Al9 | 0.314 | 0.663 | 0.032 |
| Al10 | 0.686 | 0.337 | 0.968 |
| Al11 | 0.412 | 0.464 | 0.301 |
| Al12 | 0.588 | 0.536 | 0.699 |

**Table S4**. The structural parameters of a-FeAl_2_(AF) primitive cell obtained by the structural relaxation.

| Lattice parameters |  |  |  |
| --- | --- | --- | --- |
| *a*(Å) | 4.866 | | |
| *b*(Å) | 6.464 | | |
| *c*(Å) | 8.636 | | |
| *α* | 88.728 | | |
| *β* | 74.392 | | |
| *γ* | 82.769 | | |
| Atomic coodinates | *x* | *y* | *z* |
| Fe1 | 0.137 | 0.165 | 0.416 |
| Fe2 | 0.854 | 0.839 | 0.582 |
| Fe3 | 0.233 | 0.360 | 0.866 |
| Fe4 | 0.772 | 0.637 | 0.137 |
| Fe5 | 0.989 | 0.000 | 0.995 |
| Fe6 | 0.168 | 0.474 | 0.594 |
| Al1 | 0.875 | 0.532 | 0.401 |
| Al2 | 0.493 | 0.994 | 0.162 |
| Al3 | 0.499 | 0.995 | 0.841 |
| Al4 | 0.037 | 0.116 | 0.710 |
| Al5 | 0.937 | 0.911 | 0.287 |
| Al6 | 0.589 | 0.182 | 0.541 |
| Al7 | 0.387 | 0.812 | 0.470 |
| Al8 | 0.045 | 0.286 | 0.152 |
| Al9 | 0.975 | 0.708 | 0.836 |
| Al10 | 0.300 | 0.663 | 0.041 |
| Al11 | 0.691 | 0.337 | 0.966 |
| Al12 | 0.446 | 0.424 | 0.299 |
| Al13 | 0.576 | 0.564 | 0.704 |

**Table S5**. The structural parameters of Fe_3_Al_8_-mC44 primitive cell obtained by the structural relaxation.

| lattice parameters |  |  |  |
| --- | --- | --- | --- |
| *a*(Å) | 4.959 | | |
| *b*(Å) | 6.513 | | |
| *c*(Å) | 9.970 | | |
| *α* | 99.620 | | |
| *β* | 95.296 | | |
| *γ* | 97.322 | | |
| atomic coodinates | *x* | *y* | *z* |
| Fe1 | 0.341 | 0.147 | 0.199 |
| Fe2 | 0.678 | 0.881 | 0.814 |
| Fe3 | 0.659 | 0.534 | 0.140 |
| Fe4 | 0.330 | 0.484 | 0.859 |
| Fe5 | 0.670 | 0.198 | 0.480 |
| Fe6 | 0.322 | 0.801 | 0.526 |
| Al1 | 0.976 | 0.978 | 0.035 |
| Al2 | 0.029 | 0.478 | 0.552 |
| Al3 | 0.971 | 0.204 | 0.787 |
| Al4 | 0.024 | 0.704 | 0.303 |
| Al5 | 0.158 | 0.427 | 0.086 |
| Al6 | 0.833 | 0.574 | 0.912 |
| Al7 | 0.167 | 0.108 | 0.428 |
| Al8 | 0.830 | 0.886 | 0.567 |
| Al9 | 0.450 | 0.145 | 0.691 |
| Al10 | 0.550 | 0.873 | 0.304 |
| Al11 | 0.450 | 0.808 | 0.035 |
| Al12 | 0.526 | 0.198 | 0.972 |
| Al13 | 0.474 | 0.484 | 0.367 |
| Al14 | 0.550 | 0.537 | 0.649 |
| Al15 | 0.842 | 0.254 | 0.253 |
| Al16 | 0.170 | 0.796 | 0.772 |

**Table S6.** The structural parameters of Fe_3_Al_8_-oP44 and Fe_3_Al_8_-oP44’ primitive cells obtained by the structural relaxation. The atomic coordinates of Al21, Al22, Al23, and Al24 were mainly shifted in the Fe_3_Al_8_-oP44’.

| Fe_3_Al_8_-*oP*44 | | | | Fe_3_Al_8_-*oP*44' | | | |
| --- | --- | --- | --- | --- | --- | --- | --- |
| lattice parameters |  |  |  |  |  |  |  |
| *a*(Å) | 12.401 | | |  | 12.394 | | |
| *b*(Å) | 7.617 | | |  | 7.619 | | |
| *c*(Å) | 6.392 | | |  | 6.393 | | |
| *α* | 90.000 | | |  | 90.000 | | |
| *β* | 90.000 | | |  | 90.000 | | |
| *γ* | 90.000 | | |  | 90.000 | | |
| atomic coodinates | *x* | *y* | *z* |  | *x* | *y* | *z* |
| Fe1 | 0.089 | 0.250 | 0.076 |  | 0.078 | 0.249 | 0.085 |
| Fe2 | 0.415 | 0.750 | 0.583 |  | 0.578 | 0.249 | 0.415 |
| Fe3 | 0.915 | 0.750 | 0.917 |  | 0.405 | 0.749 | 0.573 |
| Fe4 | 0.589 | 0.250 | 0.424 |  | 0.905 | 0.749 | 0.927 |
| Fe5 | 0.243 | 0.250 | 0.427 |  | 0.240 | 0.249 | 0.425 |
| Fe6 | 0.256 | 0.750 | 0.926 |  | 0.740 | 0.249 | 0.075 |
| Fe7 | 0.756 | 0.750 | 0.574 |  | 0.252 | 0.749 | 0.926 |
| Fe8 | 0.743 | 0.250 | 0.073 |  | 0.752 | 0.749 | 0.574 |
| Fe9 | 0.418 | 0.250 | 0.078 |  | 0.418 | 0.249 | 0.074 |
| Fe10 | 0.918 | 0.250 | 0.422 |  | 0.918 | 0.249 | 0.426 |
| Fe11 | 0.077 | 0.750 | 0.576 |  | 0.079 | 0.749 | 0.580 |
| Fe12 | 0.577 | 0.750 | 0.924 |  | 0.579 | 0.749 | 0.920 |
| Al1 | 0.062 | 0.250 | 0.712 |  | 0.082 | 0.249 | 0.715 |
| Al2 | 0.562 | 0.250 | 0.788 |  | 0.582 | 0.249 | 0.785 |
| Al3 | 0.419 | 0.750 | 0.214 |  | 0.445 | 0.749 | 0.215 |
| Al4 | 0.919 | 0.750 | 0.286 |  | 0.945 | 0.749 | 0.285 |
| Al5 | 0.078 | 0.054 | 0.377 |  | 0.080 | 0.048 | 0.377 |
| Al6 | 0.578 | 0.054 | 0.123 |  | 0.580 | 0.048 | 0.123 |
| Al7 | 0.418 | 0.549 | 0.876 |  | 0.418 | 0.557 | 0.880 |
| Al8 | 0.918 | 0.549 | 0.624 |  | 0.918 | 0.557 | 0.620 |
| Al9 | 0.418 | 0.951 | 0.876 |  | 0.418 | 0.942 | 0.880 |
| Al10 | 0.918 | 0.951 | 0.624 |  | 0.918 | 0.942 | 0.620 |
| Al11 | 0.578 | 0.446 | 0.123 |  | 0.580 | 0.451 | 0.123 |
| Al12 | 0.078 | 0.446 | 0.377 |  | 0.080 | 0.451 | 0.377 |
| Al13 | 0.255 | 0.059 | 0.118 |  | 0.249 | 0.071 | 0.089 |
| Al14 | 0.755 | 0.059 | 0.382 |  | 0.749 | 0.071 | 0.411 |
| Al15 | 0.245 | 0.569 | 0.597 |  | 0.240 | 0.555 | 0.623 |
| Al16 | 0.745 | 0.569 | 0.903 |  | 0.740 | 0.555 | 0.877 |
| Al17 | 0.245 | 0.931 | 0.597 |  | 0.240 | 0.944 | 0.623 |
| Al18 | 0.745 | 0.931 | 0.903 |  | 0.740 | 0.944 | 0.877 |
| Al19 | 0.755 | 0.441 | 0.382 |  | 0.749 | 0.428 | 0.411 |
| Al20 | 0.255 | 0.441 | 0.118 |  | 0.249 | 0.428 | 0.089 |
| Al21 | 0.290 | 0.250 | 0.780 |  | 0.335 | 0.249 | 0.745 |
| Al22 | 0.790 | 0.250 | 0.720 |  | 0.835 | 0.249 | 0.755 |
| Al23 | 0.174 | 0.750 | 0.256 |  | 0.218 | 0.749 | 0.288 |
| Al24 | 0.674 | 0.750 | 0.244 |  | 0.718 | 0.749 | 0.212 |
| Al25 | 0.416 | 0.076 | 0.427 |  | 0.408 | 0.069 | 0.404 |
| Al26 | 0.916 | 0.076 | 0.073 |  | 0.908 | 0.069 | 0.096 |
| Al27 | 0.086 | 0.572 | 0.912 |  | 0.078 | 0.576 | 0.929 |
| Al28 | 0.586 | 0.572 | 0.588 |  | 0.578 | 0.576 | 0.571 |
| Al29 | 0.086 | 0.928 | 0.912 |  | 0.078 | 0.923 | 0.929 |
| Al30 | 0.586 | 0.928 | 0.588 |  | 0.578 | 0.923 | 0.571 |
| Al31 | 0.916 | 0.424 | 0.073 |  | 0.908 | 0.430 | 0.096 |
| Al32 | 0.416 | 0.424 | 0.427 |  | 0.408 | 0.430 | 0.404 |

**Reference**

[1] Hinuma Y, Pizzi G, Kumagai Y, Oba F and Tanaka I Band structure diagram paths based on crystallography Comput. Mater. Sci. 2017 ;128 :140–84
